# How We Apples Do Swim

**Published:** 1900-01

**Authors:** 


HOW WE APPLES DO SWIM.—The following document was furnished the JOURNAL by the Secretary of the Quarantine Department. It is reproduced here, *verbatim.* It is from nowhere in particular it seems. All such documents go to the Quarantine Department because there is no "State Board," either of "health" or of "medical examiners," and our quarantine office is the only "medical staff" available hereabout:

Dec 15, 1899.

to the seccretary of States Medical staff Dear sir will you please inform me what it will cost for a Man to practic Medicine in your state, providing you can promit him. he tuck a corse of lectures in 96. owing to the shortage of money has bin unable to finish the other corses he has bin with me ever sens 96 and I have bin teaching him and I am fully confident that that he is able to practic Medicine in all its brantches. it will be very gratifing to me if you can admit him as he is a very promising young Man and having a Mother and Sister to mantain it would be more then pleasing to me if you can give him an opening as I have taking the greates of paines in teaching him I hope that you can admit him without any big cost beliving he will make a useful Doctor in your State as well as a fine practinear when once has a chance please let me hear from you at your earlest convenience.

DR. A. JACKSON.

For the information of "Dr. Jackson," we will state that all he has to do (as he is teaching him) is to call himself a "Faculty," and give this "Man" a diploma. That will "go" in Texas; everything "goes" here.

---

SUPERINTENDENT WORSHAM'S REPORT (Austin), Texas Insane Asylum, for the year ending October 31, 1899, has been received. Admitted, 4098; discharged, 2377; died, 911; remaining, 734. The pamphlet is adorned with full page half-tone engravings of all recent additions, including the splendid natatorium constructed under Dr. Worsham's administration. The cost of maintenance per capita was $135.92, the lowest in the history of the institutio[illegible]. The last Legislature appropriated $5500 for an artesian well at this asylum, and the well has been sunk—1900 feet deep. It is now spouting between 150,000 and 200,000 gallons of water per day. Dr. Worsham will soon cut loose from the city water plant and will utilize this splendid well, to a saving of many thousands of dollars to the institution.

Of the heavy mortality shown by the report, Dr. Worsham says: "The death rate is slightly in excess of the past year. I think this is due to the fact that last winter was the severest we have ever had almost within the history of this country. Our old and dilapidated steam plant was taxed to its utmost, but we were unable to heat the buildings comfortably, and as a result many of the old and infirm patients died in the spring and summer."

---

DR. S. M. JENKINS of Waco, a heretofore reputable physician, while on trial in the court house at Waco last month on a charge of criminal abortion, which resulted in the death of the patient, was shot in the back by a brother of the dead girl. Dr. Jenkins, it is thought, will recover. His defense is, that he found it necessary to produce abortion to save the woman's life. If there is any truth in this plea, this case should be a warning to all doctors. Cases do occur in which there is such obstinate vomiting that death by starvation or otherwise is imminent; or where, because of malformation of pelvis, or other reasons, it may become necessary to empty the uterus to save life; but no prudent man would take such a step without consultation with other physicians, and thus divide the risk, should they decide to take it; then he would have witnesses who could exonerate him from blame in case of death of the patient.

---

THE TEXAS INSANE ASYLUMS. Additions are being made to the asylums which, when completed, will accommodate 300 more patients at Terrell and 350 more at Austin. As stated elsewhere, there is accommodation now available at San Antonio for 130 additional. When these are all filled there will still be many unfortunates lingering in county jails. We have been informed by one who knows that there is considerable difficulty in getting county officials to send these patients to the asylums, because so long as they are inmates of jails they are a source of revenue to sheriffs. This should be loked into. The State owes no higher duty to humanity and to the friends and relatives of these unfortunates than that of providing at once asylum accommodations for the last one of them.

---

DR. DANIEL'S BOOK, "Recollections of a Rebel Surgeon," is now ready for delivery. Those who subscribed for a copy and did not remit at the time, are requested to forward the money, $1.10 (the ten cents for postage or expressage), and the book will be immediately sent. A copy has been reserved for each of the above mentioned orders. Of course, we could hardly be expected to send out several hundred books without the money, and have, later, to make out bills and pay postage in order to collect. We hope that no one will take offence. A dollar ten is not much, but multiplied by hundreds it is.

---

THE WASHINGTON MEETING of representatives of all the State Medical Societies in the interest of reform in sanitary legislation, and to recommend changes in the organization of the medical department of the army and navy, takes place this month. The delegates from Texas Medical Association are Prof. J. F. Y. Paine, M. D., Texas Medical College, and Dr. T. J. Tyner of Washington, D. C. Dr. H. A. West of Galveston, was chosen by the American Medical Association as the Texas representative of that body.

---

NO OCCUPATION TAX ON PHYSICIANS. Referring to Dr. Casper's article (Dental Department), we have examined the Tax Commissioner's report of the proposed change in the tax laws, and it is seen that it is not contemplated to put a State tax of $25 on physicians, as stated by Dr. Caspar. The article in the *Galveston News*, referred to by him, was a mistake on the part of the reporter. The item in the Commissioner's report applies to itinerant "doctors"—mountebank fellows.

---

DEATH OF DR. HAMMOND. Dr. W. A. Hammond, ex-Surgeon General U. S. A., and, at the time of his death, Professor of Mental Diseases in the Baltimore Medical College, died at his home in Washington City, January 5 (inst.). Dr. Hammond was one of the most famous specialists in America. He had for years been conducting a sanitarium for nervous diseases, at Washington. His career had been a checkered one—and is well known to the physicians of the United States.

---

THE TEXAS LEGISLATURE will meet this month in special session to consider, especially, a new tax bill. Efforts will be made by friends of humanity to repeal the infamous law passed last session cutting down salaries of asylum superintendents, and to take asylums out of politics; *nit*: Patronage is power, and a Governor would sooner part with an eye-tooth than let go these appointments.

---

INSANE IN TEXAS. For lack of asylum accommodation many hundred insane people are in jails in Texas. Some additions to our asylums have been made, and the Governor calls attention to the fact that 130 additional patients can be accommodated at the Southwest Texas Insane Asylum at San Antonio. This is not a drop in the bucket.

---

SUPERINTENDENT WILSON of the North Texas Insane Asylum, who accepted the appointment by request of the Governor, and who tendered his resignation when the last Legislature cut down the salaries of superintendents, insists on his resignation; and to date no one has been found to accept the position.

---

SO, THE plague has reached the Hawaiian Islands. It is knocking at the door at New York, Galveston and San Francisco, and health officers are fumigating and admitting cargoes of coffee from Santos and other infected points.

---

ATTENTION is called to the announcement in "Abstracts and

Selections," from advance sheets *Medico-Legal Journal*, of a Congress of Tuberculosis to be held in New York. The importance of this meeting and of the subject cannot be overestimated.

---

MAJOR-GENERAL LEONARD WOOD,—Governor-General of Cuba, is a doctor. Here's a case where the shoemaker got beyond his last with advantage. Dr. Wood is now immortal. He had hardly been heard of as a doctor.

---

THE Attorney-General of Missouri is now trying to get an injunction to stop Chicago from using the Mississippi river as a sewer. Humph.

---

ST. LOUIS has lost her great surgeon Dr. H. H. Mudd, nephew and successor to the great Hodgson.

---

THE JOURNAL sends greeting, and "a happy and prosperous new year" to its friends everywhere.

